# An interference account of the missing-VP effect

**DOI:** 10.3389/fpsyg.2015.00766

**Published:** 2015-06-16

**Authors:** Jana Häussler, Markus Bader

**Affiliations:** ^1^Department of Linguistics, University of PotsdamPotsdam, Germany; ^2^Department of Linguistics, Goethe University FrankfurtFrankfurt, Germany

**Keywords:** sentence parsing, center embedding, grammatical illusion, missing-VP effect, cue-based retrieval, interference, German

## Abstract

Sentences with doubly center-embedded relative clauses in which a verb phrase (VP) is missing are sometimes perceived as grammatical, thus giving rise to an illusion of grammaticality. In this paper, we provide a new account of why missing-VP sentences, which are both complex and ungrammatical, lead to an illusion of grammaticality, the so-called missing-VP effect. We propose that the missing-VP effect in particular, and processing difficulties with multiply center-embedded clauses more generally, are best understood as resulting from interference during cue-based retrieval. When processing a sentence with double center-embedding, a retrieval error due to interference can cause the verb of an embedded clause to be erroneously attached into a higher clause. This can lead to an illusion of grammaticality in the case of missing-VP sentences and to processing complexity in the case of complete sentences with double center-embedding. Evidence for an interference account of the missing-VP effect comes from experiments that have investigated the missing-VP effect in German using a speeded grammaticality judgments procedure. We review this evidence and then present two new experiments that show that the missing-VP effect can be found in German also with less restricting procedures. One experiment was a questionnaire study which required grammaticality judgments from participants without imposing any time constraints. The second experiment used a self-paced reading procedure and did not require any judgments. Both experiments confirm the prior findings of missing-VP effects in German and also show that the missing-VP effect is subject to a primacy effect as known from the memory literature. Based on this evidence, we argue that an account of missing-VP effects in terms of interference during cue-based retrieval is superior to accounts in terms of limited memory resources or in terms of experience with embedded structures.

## 1. Introduction

Some sentences are more difficult to process than other sentences, and some sentences are so complex that they exceed the processing capacity of the human parser and thereby lead to processing overload. A striking illustration of the parser's limited capacity is provided by sentences with multiple center-embedding as illustrated by the example in (1) from Frazier (1985).

(1)    The patient the nurse the clinic had hired admitted met Jack.

Sentences with multiple center-embedding have long been known to be difficult to process (Chomsky and Miller, 1963; Miller and Chomsky, 1963; Miller and Isard, 1964; Bever, 1970; Kimball, 1973, e.g.,). Sentences with two degrees of center-embedding can still be comprehended under certain conditions, as demonstrated by the following sentence from Bever (1974), in which the subject of the most deeply embedded relative clause is a first-person pronoun and not a lexical NP.

(2)    The reporter who everyone *that I met* trusts said the president won't resign yet.

Sentences with two degrees of center-embedding are also produced from time to time, at least in written language (cf. Karlsson, 2007). With two levels of center-embedding, the maximum degree of center-embedding is already reached, however, and sentences with three or more degrees of center-embedding seem to be beyond the capacity of human parsing and human sentence production.

In comparison to sentence (1), the closely related sentence in (3) seems much easier to understand.

(3)    The patient the nurse the clinic had hired met Jack.

Sentence (3) is an example of the so-called *missing-VP effect*, a term coined by Gibson and Thomas (1999) for the observation that people often fail to notice the lack of a verb phrase in sentences involving multiple center-embedding. The effect was first discussed by Frazier (1985), who attributes the observation to Janet Fodor.

Missing-VP sentences contain two degrees of center-embedding and an uncontroversial ungrammaticality. Each of these properties alone should suffice to decrease the acceptability of such sentences, and when the two properties occur together, a highly degraded sentence should result. However, instead of being perceived as highly degraded, the acceptability of such sentences is as high or even higher as the acceptability of corresponding complete and thereby grammatical sentences. This was first demonstrated by Gibson and Thomas (1999) in a rating study examining sentences like (4)^1^.

(4)    The ancient manuscript that the graduate student who the new card catalog [_VP3_ had confused a great deal]
           [_VP2_ was studying in the library] [_VP1_ was missing a page].

Sentences were either complete or were missing one of VP1, VP2, or VP3 and had to be rated for their intuitive complexity. While sentences with either missing VP1 or missing VP3 were rated as being significantly more complex than complete sentences, the ratings for sentences with missing VP2 did not differ significantly from the ratings for complete sentences. Later research by Christiansen and MacDonald (2009) and Vasishth et al. (2010) showed that sentences in which VP2 is missing are more often perceived as grammatical and are easier to process than corresponding complete sentences with two degrees of center-embedding. Gimenes et al. (2009) have found similar results for French, another SVO language. The only SOV language for which evidence on the missing-VP effect exists seems to be German, but this evidence is mixed. Since our experiments investigate German sentences, we postpone a discussion of the missing-VP effect in this language to Section 4.

The missing-VP effect belongs to a small class of grammatical illusions—sentences which tend to be perceived as grammatical despite containing an undisputed ungrammaticality. In their review of grammatical illusions, Phillips et al. (2011, p. 166) exclude the missing-VP effect from further consideration because examples as in (3) “differ from the others discussed here in the respect that they plausibly reflect complexity-induced overload, and it is not clear what parse is assigned to such dramatically ill-formed sentences”. We take the view that the missing-VP effect reflects complexity-induced overload to be uncontroversial. However, there are competing conceptions as to the source of parsing overload. The major aim of this paper is to provide an account of the missing-VP effect that follows much recent work in cognitive psychology and psycholinguistics claiming that overload is mainly a matter of interference during memory retrieval. Based on this hypothesis, we will argue that the parse assigned to missing-VP sentences differs minimally from the parse assigned to corresponding complete sentences. The only difference is that for complete sentences, all VP slots of the syntactic representation are filled by lexical material whereas for missing-VP sentences one of the VP slots remains empty. These claims are based on a review of prior experimental investigations of the missing-VP effect in German and on two new experiments, which were run with the additional aim of resolving some contradictions that concern the status of the missing-VP effect in German.

The organization of this paper is as follows. In Sections 2 and 3, we discuss two approaches to capacity limitations of cognitive processes and how they might account for the missing-VP effect. Section 2 introduces the resource account of capacity limitations and Section 3 the interference account. Section 4 reviews evidence from German favoring the interference account over the resource account. Some concerns regarding this evidence is addressed by two experiments that are presented in Sections 5 and 6. Section 7 concludes with a general discussion of the experimental results.

## 2. Resource accounts of the missing-VP effect

The parser is not alone in being capacity limited. Most if not all cognitive abilities share this property. For example, our ability for mental calculations is restricted to a small subset of numbers, our ability to recall lists of unrelated items is limited to lists of no more than seven or eight items, and so on. Limitations of this kind are often attributed to a working memory system of limited capacity. The question then becomes why working memory has such a severely limited capacity. Over time, this question has received various answers (see overviews in Oberauer and Kliegl, 2001; van Dyke and Johns, 2012).

Before we take a closer look at these answers, let us first get clear about the tasks that have to be accomplished in order to parse sentences successfully. By definition, a parser takes the words of an input string and constructs a syntactic structure for them. In the following, we assume the syntactic structure to be a conventional phrase-structure tree. Given the strong evidence that human parsing proceeds in an incremental way, the parser's task can be divided into two major subtasks (see Just and Carpenter, 1992; Gibson, 1998). First, the parser must store the syntactic structure, which is incremented word-by-word, in some kind of temporary buffer. Secondly, the parser must integrate each word of the input string into the unfolding syntactic structure as soon as the word is encountered. This subtask can be decomposed further. First, the parser must find a place within the ongoing syntactic structure where the word can be attached. Second, the word must be connected to words that are already part of the ongoing syntactic structure. For example, a verb must be connected to its arguments for thematic role and case assignment and for checking agreement requirements.

With the distinction between storage and integration at hand, we now come back to the question of why human parsing is subject to severe capacity limitations. For a long time, the dominant approach to capacity limitations was based on the claim that cognitive processes draw on a limited pool of processing resources. Applied to the issue of sentence parsing, the Resource Hypothesis states that the parser can use only a fixed amount of resources for the storage and computation of syntactic structures. When the available resources do not suffice for processing sentences of high complexity, processing overload results. An influential theory of sentence comprehension building on the Resource Hypothesis is the Capacity Theory of Just and Carpenter (1992). According to the Capacity Theory, each individual has a fixed amount of processing resources available for processing language. These resources can be allocated flexibly to the storage of intermediate syntactic structures and the incremental integration of words into the intermediate structure built thus far. The assumption that the parser must use a fixed pool of resources for both storage and processing is shared by the Syntactic Prediction Locality Theory (SPLT) of Gibson (1998) and its successor, the Dependency Locality Theory (DLT) of Gibson (2000).

According to resource-based theories, sentences with a high degree of center-embedding cannot be successfully parsed because they require more resources than are available. This suggests an explanation of the missing-VP effect along the following lines. When the parser is processing a sentence of high syntactic complexity, it may be short of running out of resources. In such a case, the parser can try to proceed by forgetting some part of the structure built thus far, thereby freeing resources needed to continue the ongoing parsing process. Two implementations of this idea are the *Disappearing Syntactic Nodes Hypothesis* of Frazier (1985) and the *High Memory Cost Pruning Hypothesis* of Gibson and Thomas (1999). Both implementations share the idea that the phrase structure tree is cut down under conditions of high memory load. For reasons of space, we only give the High Memory Cost Pruning Hypothesis below.

(5)    *The High Memory Cost Pruning Hypothesis* (Gibson and Thomas, 1999, p. 231)
          At points of high memory complexity, forget the syntactic prediction(s) associated with the most memory load.

The High Memory Cost Pruning Hypothesis was formulated within the SPLT of Gibson (1998). According to the SPLT's definition of storage cost, the prediction of VP2 is associated with the most memory load in sentences with doubly center-embedded relative clauses (see Gibson and Thomas, 1999; Vasishth et al., 2010, for details). The prediction of VP2 is therefore forgotten. Instead of the complete tree shown on the left side in (6), the incomplete tree on the right side is available for the parser at the point where the two final VPs of a missing-VP sentence are about to be integrated.

The first VP that the parser encounters is put into the open slot for VP3 and the next VP into the slot for VP1. In a sentence with missing VP2, all VPs of the input string can thus be successfully integrated. Because the slot for VP2 is no longer present in the syntactic representation, the parser fails to notice the lack of a VP. In the case of complete sentences with a doubly center-embedded relative clause, the representation on the right side of (6) does not provide an attachment site for each VP. Such sentences should

(6) 
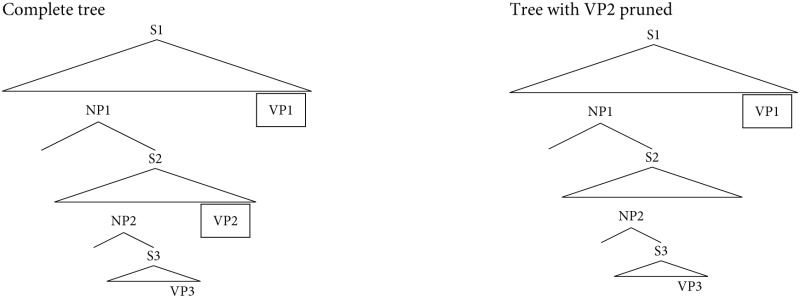

thus be more difficult to process than sentences with a missing VP. This was not the case in the off-line ratings reported in Gibson and Thomas (1999), but on-line evidence obtained by Christiansen and MacDonald (2009) and Vasishth et al. (2010) shows that sentences in which VP2 is missing are easier to process than corresponding sentences in which VP2 is present.

A conceptual drawback of the High Memory Cost Pruning Hypothesis is that it does not follow from independently motivated principles of storage or computation. Resource-based accounts of capacity limitations typically assume that trace decay is an important source of storage limitations. When applied to sentences with double center-embedding, the prediction of VP1

(7)    [_S1_ [*The reporter*] [_S2_ who [**everyone**] [_S3_ that I       [_V3_ met] ] [_V2_ **trusts**] …
          [_S1_ NP1                [_S2_         NP2           [_S3_        NP3               ]

should be less available than the prediction of VP2 because it was introduced earlier and had therefore more time for decay. This is just the opposite of what the Pruning Hypothesis claims and is accordingly not compatible with the findings for missing-VP sentences. Additional machinery is therefore necessary in order to derive that the prediction of VP2 is pruned but not the prediction of VP1. For example, the parser must somehow be able to calculate the memory load of each prediction in order to prune the one with the most memory load. These calculations are heavily theory dependent. As discussed in more detail in Vasishth et al. (2010), the memory-load definitions of the DLT do no longer predict that VP2 is pruned, although—as also shown in Vasishth et al. (2010)—it is possible to adapt the Pruning Hypothesis to the particular properties of the DLT.

We will not dwell further on this issue because resource-based accounts of capacity limitations in general and the concept of trace decay have fallen into disreputation, both for theoretical reasons (e.g., Navon, 1984, MacDonald and Christiansen, 2002) and for lack of empirical support (e.g., Oberauer and Kliegl, 2001). Two influential alternatives to the resource-based view are the *interference account* and the *experience-based account* (further alternatives are discussed in Oberauer and Kliegl, 2001). In the next section, we propose an explanation of the missing-VP effect that is based on the interference account. The experience-based account is discussed in the final section^2^.

## 3. An interference account of the missing-VP effect

The interference account is based on the observation that the retrieval of material from working memory becomes less reliable in the presence of similar material. In order to apply the interference account to the process of sentence parsing, we have to take a closer look at the steps that lead to the integration of new words into the unfolding syntactic representation. A crucial first step for successful integration is the retrieval of the correct attachment site for the word that is to be integrated. If the retrieval cues used for this purpose match more than a single attachment site, finding the proper place for attaching the next word can become more difficult.

While interference from similar items can make the integration of new items more difficult, whether interference does indeed occur depends on the particular syntactic configuration. Consider first the situation that obtains in sentences with double center-embedding at the point where the verb of the most deeply embedded relative clause has to be integrated, that is, *met* in sentence (7).

The syntactic representation built up to this point contains three clauses which still need a VP, namely the matrix clause S1 and the two relative clauses S2 and S3. Despite the existence of three potential attachment sites, the integration of the verb *met* into the embedded relative clause will not be disturbed by the presence of other potential attachment sites. The reason for this is that the most recently read subject is in the focus of attention and therefore immediately available for integration (see McElree, 2006, for the notion of focal attention as used in the memory literature).

The situation changes when the parser encounters the verb of the higher relative clause, *trusts* in sentence (5). After the most deeply embedded relative clause has been processed, the ongoing phrase-structure representation still contains two possible attachment sites for a verb. In this case, choosing the correct attachment site is not so easy for the parser because due to the intervening relative clause S3, the parser faces the task of switching back to a clause that is no longer in the focus of attention. Because there are two such clauses and each contains a slot for a VP, retrieving the correct integration site is difficult due to interference from the competing integration site.

Experimental evidence that attaching a word into the current clause is qualitatively different from attaching it to a clause that has been interrupted by one or more embedded clauses has been provided by McElree et al. (2003). In an experiment using a response-signal speed-accuracy tradeoff procedure, the verb either occurred adjacent to the head noun of the subject NP or was separated from it by either one or two relative clauses plus an additional PP in some cases. The results show that sentences in which the verb was adjacent to the subject head noun were associated with a higher asymptotic accuracy and also with a faster retrieval speed. This suggests that integrating a word with the immediately preceding word has a special status, in accordance with findings from the memory literature that only the most recent item is in focal attention (see McElree, 2006). However, Foraker and McElree (2011) and McElree and Dyer (2013) cite unpublished data by McElree and Wagers that argue against a too narrow definition of focal attention for the purpose of sentence parsing. McElree and Wagers found that an intervening relative clause removes the subject head noun from focal attention but an intervening PP (8a) or an intervening adverbial (8b) do not.

(8)    a.   The crowd gasped as the driver *of the ambulance* fainted.
          b.   The crowd gasped as the driver *abruptly* fainted.

At the current state of knowledge, it does not seem to be possible to come up with a precise definition of the scope of focal attention. What can be concluded from the literature is that an intervening relative clause removes material preceding it from focal attention whereas at least some non-clausal constituents do not. We therefore propose the *Discrimination Hypothesis* in (9) (for related ideas, see Bader et al., 2003; Lewis and Vasishth, 2005; and Bader, 2015).

(9)    The *Discrimination Hypothesis*
  The integration of new material becomes difficult when an intervening clause separates the word that is to be integrated next from the required integration site and an incorrect but similar integration site competes for attachment.

When processing a sentence with doubly center-embedded relative clauses, two potential integration sites are available at the point where the second verb has to be integrated into the ongoing syntactic representation. According to the *Discrimination Hypothesis*, these two integration sites are difficult to discriminate. When the second verb has to be integrated, the parser may therefore retrieve S1 as integration site for V2 instead of S2. This will result in a syntactic representation in which the verb slot of S1 is filled whereas the verb slot of S2 remains empty. Because complete sentences exhibiting double center-embedding and corresponding missing-VP sentences diverge only after the second verb, such an ill-formed representation can arise for both of them. A mis-attachment of V2 to the verb slot of S1 can therefore happen in both cases. In complete sentences, this can give rise to the well-known processing difficulties of sentences with double center-embedding. For missing-VP sentences, this can lead to an illusion of grammaticality.

Crucially, the Discrimination Hypothesis does not claim that anything is deleted from the ongoing syntactic representation of a sentence. The only claim is that the parser sometimes attaches a word to an incorrect attachment site. As a result, the syntactic structure for a sentence can contain a node that has not been filled with lexical material. In order for a grammatical illusion to occur, the parser must not detect that a VP slot is still empty after the last word of the sentence has been processed. We therefore have to complement the Discrimination Hypothesis with appropriate assumptions concerning the processes that check whether a sentence obeys all syntactic constraints or not^3^.

What could be the reason that the parser at times fails to detect that a sentence is incomplete? To begin with, consider sentence (10), a variant of sentence (4) of Gibson and Thomas (1999).

(10)    [_S1_ A page was missing in the ancient manuscript
  [_S2_ that the graduate student [_S3_ who the new card catalog had confused a great deal]]].

In contrast to the original example, the higher relative clause appears in a sentence final position in (10) and is thus no longer center-embedded. In sentence (10), VP2 (the VP of the higher relative clause) is missing, but in this case the resulting ungrammaticality seems easy to detect^4^. This shows that a missing VP goes unnoticed only under conditions of high processing load. The question then is what these conditions are. One major issue concerns the absence of a grammatical illusion when VP1 is missing, that is, in sentences as in (11) [partially repeated from (4)].

(11)    The manuscript that the graduate student who the new card catalog had confused a great deal *was studying in the library*.
  a.    Integration of the final verb *was studying* as VP1 into S1:
  [_S1_ *The manuscript* [_S2_ that the graduate student [...] Δ] *was studying in the library*.]
  b.    Integration of the final verb as VP2 into S2:
  [_S1_ The manuscript [_S2_ *that the graduate student* [...] *was studying in the library*.]Δ]

Sentences of this type were rated as highly complex in the experiment of Gibson and Thomas (1999). There are at least two alternative reasons for this. First, the final VP in (11) is integrated into S1 as VP1, giving rise to the configuration in (11a), which is complex due to its semantic implausibility. Alternatively, the final VP could be integrated into S2 as VP2, resulting in the configuration in (11b). This configuration will be perceived as complex only when one detects that the initial NP remains without a VP.

According to the Discrimination Hypothesis, VP2 and VP1 are both available as attachment sites when the final VP in (11) is about to be integrated. The finding of Gibson and Thomas (1999) that a missing-VP effect occurs when VP2 is missing but not when VP1 is missing can be accounted for in an interference-based framework by recourse to the notion of primacy. The opposite notion, namely recency, has already been made responsible for the fact that integrating V3 into S3 is not subject to interference and therefore unproblematic. S1 in sentences like (11) is the first clause not only in a hierarchical sense but also in a temporal sense. It therefore enjoys the advantage of primacy that is well-known from studies of memory retrieval (e.g., Knoedler et al., 1999). This advantage can have two consequences. First, it can cause V2 to be integrated more readily into S1 than into S2, resulting in configuration (11a). Second, it can ease the detection of a missing VP1 in case V2 was correctly integrated into S2, as in (11b). If this reasoning is on the right track, it should be possible to find evidence for a missing-VP effect for VP1 when the primacy advantage is taken away from S1. Evidence of this kind is discussed in the next section.

## 4. Evidence for the interference account

Experimental evidence for the interference account presented in the preceding section comes from an investigation of the missing-VP effect in German by Bader et al. (2003). In contrast to Gibson and Thomas (1999), who had participants rate the complexity of sentences on a scale from 1 to 5, Bader et al. (2003) required participants to give a binary grammaticality judgment. The rationale behind this decision was as follows. The defining property of a grammatical illusion is that a sentence is perceived as grammatical despite containing an undisputed ungrammaticality. Thus, the most straightforward way to test whether an ungrammatical sentence causes a grammatical illusion or not is to have native speakers judge its grammaticality. If the sentence is judged as grammatical, we can conclude that it caused a grammatical illusion.

Things are more complicated because we cannot expect that the illusion of grammaticality will arise on each single occasion. For some of the grammatical illusions that are discussed in Phillips et al. (2011), judgment data are available, showing that the strength of such illusions can vary considerably. For example, sentences with a negative polarity item and a negation not c-commanding the polarity item give rise to a negative polarity illusion. In a judgment experiment by Drenhaus et al. (2005), such sentences were erroneously accepted as grammatical in 30% of the time, which is only 10% more than for sentences with a negative polarity item and no negation at all. For the case illusion reported in Bader et al. (2000) and Meng and Bader (2000), the false acceptance rate was about 40% for sentences in which the verb assigned dative case to an NP which was case-ambiguous but not compatible with dative case (Bader et al., 2000). When this NP was made more complex by adding a relative clause, the false acceptance rate increased to a value of about 60% (Meng and Bader, 2000). Grammatical illusions are thus not an all-or-nothing matter, but a probabilistic phenomenon instead. Grammatical illusions do not differ from semantic illusions in this respect. For example, when testing the Moses illusion by means of a truth judgment task, Erickson and Mattson (1981) found that the sentence *Moses took two animals of each kind on the Ark*. was judged as true by 41% of the participants who possessed the relevant knowledge. Thus, semantic illusions are probabilistic too.

The particular procedure for obtaining grammaticality judgments used by Bader et al. (2003) was the procedure of speeded grammaticality judgments. Sentences were presented visually one word at a time. Participants were asked to judge sentences as either grammatical or ungrammatical as quickly as possible. A time limit of 2000 ms starting at the offset of the last word was imposed in order to encourage fast decisions. On average, participants responded even faster. Using this method, Bader et al. (2003) investigated whether the evidence provided by Gibson and Thomas (1999) can be replicated for German. The experiments provided two major results. First, participants accepted sentences with a missing VP as grammatical in a substantial number of trials. This shows that the grammatical illusion caused by a missing VP also occurs in German—at least when participants have to judge sentences under time pressure. The second major finding concerns the difference between sentences in which VP1 is missing and sentences in which VP2 is missing. In accordance with the initial observation in Frazier (1985), Gibson and Thomas (1999) found that the missing VP effect occurs when VP2 is missing but not when VP1 is missing. The same was found for German sentences which were similar to the sentences investigated by Gibson and Thomas in that the head noun of the highest relative clause was part of a main clause. When this noun was part of an embedded that-clause, however, participants often accepted incomplete sentences whether VP1 or VP2 was missing.

Thus, missing-VP sentences in which the final VP was syntactically and semantically compatible with attachment to either S1 or S2 were accepted most of the time. A relevant example is provided in (12).

(12)    *Missing VP, singular S2 subject*
                         Klaus      hat       mir     erzählt,
                         K.           has      me      told
        S1                 dass      **jemand**           die     Sängerin,
                              that      someone.sg      the    singer.sg
        S2                          *die*            den     Moderator,
                                      who.sg      the      moderator
        S3                                 der       das      Interview       trotz       einer       Grippe       führen       musste,
                                              who     the       interview       despite    a            flu             conduct     must
        S1/S2                    ***beleidigt***     ***hat***.
                                     insult            has.sg
  *final verb in S2*:   “Klaus told me that someone Δ the singer who insulted the moderator who had to conduct the interview despite a flu”
  *final verb in S1*:   “Klaus told me that someone insulted the singer who Δ the moderator who had to conduct the interview despite a flu”

In (12), a verb with an animate subject and an animate direct object is required for completion of both S1 and S2. Since the clause-final verb *beleidigt hat* (“insulted has”) meets both requirements, it can be attached to either S1 or S2. For such sentences, the acceptance rate reached a high value of about 75%, which is even slightly higher than for complete sentences. When syntactic or semantic constraints only allowed attachment to either S1 or S2, missing-VP sentences were accepted significantly less often, although still about half of the time. In the context of other types of grammatical illusions, missing VPs thus give rise to a rather strong illusion.

There is a caveat, however. Grammaticality judgments provide the most direct way of testing whether participants experience a grammatical illusion, but they are not without problems. This holds in particular when judgments must be given under time pressure, as in the experiments of Bader et al. (2003). Without further evidence, it cannot be excluded that the grammatical illusion found by Bader et al. was caused by the strict timing conditions imposed by the procedure of speeded grammaticality judgments. In order to address this issue, Experiment 1 replicates one experiment of Bader et al. (2003) using a judgment procedure that neither limits the time to process a sentence nor the time for giving a judgment.

An even more serious issue was brought about by Vasishth et al. (2010) who investigated the missing-VP effect in both English and German by recording reading times. A German example from Vasishth et al. (2010) is shown in (13).

(13)    [_S1_ Der Anwalt, [_S2_ den der Zeuge, [_S3_ den   der Spion [_VP3_ betrachtete,]] [_VP2_ schnitt,]] [_VP1_ überzeugte den Richter.]]
                  the  lawyer         who the witness      who  the spy            watched                avoided         convinced  the  judge
  “The lawyer that the witness that the spy watched avoided convinced the judge.”

The study included complete sentences as in (13) as well as incomplete sentences in which the intermediate verb [= *schnitt* in (13)] was missing. In a self-paced reading experiment and in an eye-tracking experiment, Vasishth and colleagues found longer reading times for the last verb (*überzeugte*) and the following NP in incomplete sentences compared to complete sentences. For English, in contrast, Vasishth and colleagues found the opposite pattern. Reading times for the last verb were longer in complete sentences than in incomplete sentences. The authors take the reading time increase in German as indicating that their participants noticed the ungrammaticality. Based on the crosslinguistic difference between German and English, Vasishth et al. conclude that the German reader's parser is more adapted to keeping track of upcoming verbs due to the verb-final nature of German.

Using again a speeded grammaticality judgment procedure, Bader (2015) found evidence for a missing-VP effect in sentences structurally similar to those investigated by Vasishth et al. (2010). When taking the whole literature into account, we arrive at the generalization that a missing-VP effect was found for German when using the method of speeded grammaticality judgments but not when using reading time measures. Experiment 2 therefore uses a self-paced reading procedure that does not require any grammaticality judgments at all in order to test whether the missing-VP effect also occurs under more natural reading conditions for the kind of sentences for which only judgment data are available so far.

## 5. Experiment 1

Experiment 1 has two aims. The first aim concerns the question of whether the illusion of grammaticality caused by missing-VP sentences also occurs when participants are not set under time pressure. To answer this question, Experiment 1 obtained grammaticality judgments without time limits on either reading a sentence or judging its grammaticality.

If the missing-VP effect is indeed independent of time constraints on reading and judging a sentence, the next question is whether we can replicate the finding of Bader et al. (2003) that a grammatical illusion can arise not only when VP2 is omitted but also when VP1 is omitted. In the prior literature, sentences with a missing VP1 were rarely investigated after Gibson and Thomas (1999) found that such sentences are rated as highly complex. In Bader et al. (2003), the missing-VP1 effect was restricted to sentences in which S1 is an embedded clause. The second aim of Experiment 1 is therefore to examine whether a missing-VP1 effect arises and whether it depends on the clause type of the corresponding S1.

To test this question, Experiment 1 adopts the design and materials of Experiment 2 in Bader et al. (2003). Experiment 1 varies the clause type of S1 such that S1 is either an embedded complement clause as in (14) or a main clause as in (15). In addition, the experiment varies whether VP1 or VP2 is omitted as indicated in (14) and (15) by crossing.

Two subprocesses within the human parser are crucially involved when sentences with a missing VP elicit a grammatical illusion. First, either S1 or S2 is retrieved as integration site for the final VP. Second, the resulting structure is accepted as grammatical despite the lack of a VP. When S1 is selected for integration, a missing-VP effect arises when the lack of VP2 goes unnoticed. When S2 is selected for integration, a missing-VP effect arises when the lack of VP1 goes unnoticed. The clause type of S1 could influence both the likelihood of retrieving the wrong attachment site and the likelihood of noticing the lack of a VP. It will thereby determine the probability that a missing-VP effect is observed.

Why should clause type of S1 matter? When S1 is a main clause, it is the first clause and might benefit from primacy effects as observed in the literature on memory retrieval (for a recent overview, see Knoedler et al., 1999). Adding a level

(14)   *Relative clauses within a complement clause*
Ich   habe   gehört
I      have   heard
S1         dass   seit       heute    Mittag    die    Praktikantin,
              that    since     today   noon       the    intern
S2                 die       den   Systemabsturz,
                      who     the    system_crash
S3                          der        die   Technikerin      für   etliche    Stunden    beschäftigt    hatte,
                              which    the   engineer           for   several  ours          occupied       had
S2                  [_VP2_ *verursacht*    *hat*],
                              caused          has
S1         [_VP1_    *verschwunden*        *ist*.]
                      disappeared.ptcp    is
*Complete*:        “I have heard that the intern who caused the system crash which occupied the engineer for several hours disappeared since noon.”
*Missing VP2*:   “I have heard that the intern who Δ the system crash which occupied the engineer for several hours disappeared since noon.”
*Missing VP1*:   “I have heard that the intern who caused the system crash which occupied the engineer for several hours Δ since noon.”

(15)    *Relative clauses within a main clause*
S1          Seit       heute   Mittag   ist    die   Praktikantin,
              Since     today   noon    is      the   intern
S2                    die     den   Systemabsturz,
                        who   the    system_crash
S3                            der     die   Technikerin   für   etliche   Stunden   beschäftigt   hatte,
                                who    the  engineer        for   several  hours       occupied     had
S2                     [_VP2_ *verursacht*   *hat*],
                                caused          has
S1            [_VP1_ *verschwunden*.]
                       disappeared.ptcp
*Complete*:     “Since noon, the intern who caused the system crash which occupied the engineer for several hours is missing.”
*Missing VP2*:   “Since noon, the intern who Δ the system crash which occupied the engineer for several hours is missing.”
*Missing VP1*:   “Since noon, the intern who caused the system crash which occupied the engineer for several hours is Δ.”

of embedding changes the accessibility of S1. As an embedded clause, S1 is no longer the first clause but occurs in an intermediate position in the sequence of clauses. S2, in contrast, occurs always in an intermediate position between at least two clauses, namely S1 and S3 regardless of the type of S1. A primacy advantage of S1 in sentences in which S1 is a main clause could affect the processing of missing-VP sentences in two ways: First, it might increase the probability of integrating the final VP into S1 and thus decrease the probability of integrating it into S2. Second, in case the final VP was integrated into S2, the primacy advantage might increase the probability of detecting that S1 is missing a verb. These two possible consequences of the increased salience of S1 do not exclude each other. Both could jointly prevent a missing-VP effect in sentences in which S1 is a main clause and VP1 is missing. In these sentences, integration of VP2 into S1 results in a syntactic and semantic conflict, which prevents the illusion of grammaticality. Attachment to S2 would leave S1 with a missing VP, which will be noted thanks to the salience of S1. Thus, primacy predicts that the likelihood of a grammatical illusion in missing-VP2 sentences depends on the level of embedding of S1.

In missing-VP2 sentences, a grammatical illusion arises when the remaining VP1 is correctly integrated into S1 and the lack of a VP in S2 goes unnoticed, or when VP1 is integrated into S2 and the lack of a VP in S1 goes unnoticed. Primacy effects might increase the chance of S1 integration and thereby increase the likelihood of a grammatical illusion (under the assumption that the likelihood of detecting a missing VP in S2 is independent of the status of S1). But at the same time, primacy would increase the chance of detecting a missing VP in S1 when VP2 is correctly integrated into S2. Taken together, primacy predicts a lower rate of grammatical illusions in sentences in which S1 is a main clause and VP1 is missing.

### 5.1. Method

#### 5.1.1. Participants

Twenty-four students at the University of Konstanz participated in Experiment 1. In this and the following experiment, all participants were native speakers of German and were naive with respect to the purpose of the experiment. They were either paid or received course credit for participation in the experiment.

#### 5.1.2. Materials

The materials for Experiment 1 consisted of 30 sentences that were taken from Bader et al. (2003). Each sentence appeared in six versions according to the two factors Clause Type and Structure. The factor Clause Type varied the type of the matrix clause of the higher relative clause. This was either an embedded complement clause as in (14) or a main clause as in (15). The factor Structure manipulated whether the sentence was complete or not. In case it was not complete, either VP2 or VP1 was missing as indicated in (14) and (15) by crossing.

Sentences in the condition “main clause” consisted of three clauses: a main clause (S1), a relative clause (S2) center-embedded into the main clause, and a second relative clause (S3) center-embedded into the first relative clause. All main clauses started with an adverbial followed by the finite auxiliary and the subject NP. This subject NP was modified by the first relative clause. This relative clause was a subject-initial relative clause whose object NP was modified by the second relative clause. Each relative clause ended in a lexical verb followed by an auxiliary whereas the main clause ended in a lexical verb only because the main-clause auxiliary occurred already in the second position of the sentence. Sentences in the condition “embedded clause” contained one more level of embedding and thus consisted of four clauses: a short main clause, a complement clause (S1) and two center-embedded relative clauses (S2 and S3). The short main clause always preceded the complement clause. In complete sentences, all three verbs were present. In missing-VP sentences, either VP2 (lexical verb and auxiliary) or VP1 [lexical verb and auxiliary in the condition “embedded clause” and just lexical verb in the condition “main cause,” in which the auxiliary appeared in the main clause, cf. (15)] was missing. The lexical verbs in VP1 and VP2 were always compatible with an animate subject and insofar compatible with both S1 and S2. However, their syntactic properties prevent them from being interchangeable: V1 was an intransitive verb while V2 was transitive.

The sentences were distributed across six lists using a Latin square design. Each list contained only a single version of each sentence and an equal number of sentences in each condition. The experimental lists were interspersed in a list of about 260 filler sentences for Experiment 1. The majority of filler sentences was from unrelated experiments. Each participant saw only one list.

#### 5.1.3. Procedure

Participants received a questionnaire on which the experimental sentences were printed. They were asked to judge the grammaticality of each sentence on the questionnaire by marking one of the two options “grammatical” or “ungrammatical” printed beneath each sentence. Participants could spend as much time as they wanted on reading the sentences and giving their judgments. On average, they needed about 45–50 min to complete the questionnaire.

### 5.2. Results

For each participant and item, we recorded the grammaticality judgment. Table 1 shows the results in terms of acceptance rates. All statistical analyses reported in this paper were computed using the statistics software R, version 2.14.2 (R Development Core Team, 2012). Responses were analyzed by means of linear mixed-effects logistic regression using the R-package *lme4* (Bates and Maechler, 2010). Forward difference coding was used for the experimental factors. That is, they were coded in such a way that all contrasts tested whether the means of adjacent factor levels were significant. Contrasts were specified as follows. For the factor Clause Type, the mean results in the condition “main clause” are contrasted with the mean results in the condition “embedded clause.” For the factor Structure, two contrasts were defined. The first one compares complete sentences to sentences with a missing VP2 and the second one compares sentences with a missing VP2 to sentences with a missing VP1. Since not all possible contrasts can be tested within one model, we chose the contrasts such that the condition with the highest acceptance rates (complete sentences) is compared to the condition with intermediate acceptance rates (missing VP2), which in turn is compared to the condition with the lowest acceptance rates (missing VP1). If both contrasts turn out to be significant, we can conclude that the remaining contrast (complete sentences vs. sentences missing VP1) is significant as well. We included participants and items as crossed random effects. Following the advice given in Barr et al. (2013), we first computed a model containing the full factorial design in the random slopes. Since this model did not converge, we dropped the interaction term from the random sentence factor, which resulted in a converging model. For each contrast, Table 2 shows the estimate, the standard error, the resulting *z*-value and the corresponding *p*-value.

**Table 1:** **Acceptance rates in Experiment 1**.

	**Complete**	**Missing**	**Missing**
		**V2**	**V1**
Main clause	81 (5.6)	33 (7.1)	10 (4.0)
Embedded clause	81 (5.4)	41 (8.5)	33 (7.5)

*Standard error (by participants) is given in parentheses*.

**Table 2:** **Mixed-effects model for the judgment results of Experiment 1**.

**Contrast**	**Estimate**	**Std. error**	***z*-value**	**Pr(>|*z*|)**
Clause Type	−3.585	0.834	−4.297	< 0.001
Structure 1	−1.311	0.524	−2.502	< 0.05
Structure 2	0.903	0.435	2.083	< 0.05
Structure 1 × Clause Type	0.419	0.721	0.581	0.562
Structure 2 × Clause Type	1.834	0.771	2.377	< 0.05

The factor Clause Type was significant, with sentences in the condition “embedded clause” being judged as grammatical more often than sentences in the condition “main clause” (52 vs. 41%). The two contrasts of the factor Structure were also significant. Complete sentences received higher acceptance rates than missing-VP2 sentences (81 vs. 37%) which in turn received higher acceptance rates than missing-VP1 sentences (37 vs. 22%). Of the two interactions, only the one involving the second contrast of the factor Structure was significant. This reflects the finding that for complete and missing-VP2 sentences, the factor Clause Type did not have much of an effect whereas for missing-VP1 sentences, sentences in the condition “embedded clause” were more often accepted as grammatical than sentences in the condition “main clause.”

Pairwise comparison were computed in order to explore the interaction more closely. Sentences with a missing VP1 received significantly fewer grammatical judgments than sentences with a missing VP2 when S1 was a main clause (33 vs. 10%, *z* = 4.71, *p* < 0.001). In sentences with an embedded S1, in contrast, the difference between missing VP2 and missing VP1 was not significant (41 vs. 33%, *z* = 1.49, *p* = 0.14). Furthermore, sentences with a missing VP1 were judged as grammatical significantly less often when S1 was a main clause than when S1 was an embedded clause (10 vs. 33%, *z* = 4.82, *p* < 0.001). For sentences with a missing VP2, the contrast between main and embedded S1 clause was marginally significant (33 vs. 41%, *z* = 1.65, *p* = 0.10).

### 5.3. Discussion

Experiment 1 has yielded two major results. First of all, although participants had unlimited time for reading and judging a sentence, sentences in which a VP was missing were accepted as grammatical in a substantial number of cases. Though the observed missing-VP effects were somewhat weaker in the current experiment than in the corresponding speeded grammaticality judgment experiment from Bader et al. (2003), the questionnaire results closely replicate the pattern from the speeded grammaticality judgments study (correlation coefficient for grand means: *r* = 0.94, *p* < 0.01; for items means per condition: *r* = 0.31, *p* < 0.001). Moreover, the average acceptance rate for missing-VP sentences in the questionnaire study was still 29% despite the lack of time pressure. The mean acceptance rate was even higher when we excluded main clauses with a missing VP1. For these sentences, no missing-VP effect was expected, and in accordance with this expectation, they were rejected as ungrammatical in about 90% of the time. The finding that the other missing-VP sentences are accepted as grammatical to a substantial degree despite the lack of time constraints corroborates the existence of the missing-VP effect in German. Given the interaction of Clause Type and Structure, the missing-VP effect cannot be attributed to an undifferentiated tendency to accept sentences of this type as grammatical. In sum, participants experience a grammatical illusion with missing-VP sentences not only when put under time pressure, but also when they have as much time as they want. The possibility to reread sentences and to engage in deliberate reasoning reduces the missing-VP effect, but it does not eliminate it.

The second major finding of Experiment 1 is that a missing-VP effect for VP2 is independent of clause type whereas a missing-VP effect for VP1 is restricted to sentences in which S1 is an embedded clause. Sentences lacking a VP in their main clause were reliably rejected as ungrammatical with a 90% rejection rate. This finding is compatible with the proposal that primacy effects make it easier to spot the lack of a VP in the first clause, i.e., the main clause, of a complex sentence.

For sentences with a missing VP2, clause type had no effect. The lack of a difference between main and embedded clauses indicates that properties of S1 did not affect the probability of detecting that VP2 was missing. This is expected under the primacy perspective since S2 is always an embedded clause. Promoting S1 to a main clause brings S1 into first position but leaves S2 in an intermediate position.

The clause type of S1 had also no effect for complete sentences. The finding of identical acceptance rates for main and embedded matrix clauses confirms earlier claims that clausal embedding does not cause increased processing costs as long as clauses are embedded in sentence final position (see Gibson, 1998; Gibson and Thomas, 1999). Erroneous integration of VP2 or VP1 into the wrong clause might occur from time to time but is easily detected because of the other VP. If VP2 is erroneously attached to S1, the subsequent verb (VP1) signals the error. An attempt to attach VP1 to S2 will fail because the verb slot of S2 is already occupied by VP2.

In sum, Experiment 1 has shown that the grammatical illusion caused by a missing VP2 is a robust phenomenon which is not affected by whether S1 is a main clause or an embedded clause. A missing VP1, in contrast, causes a grammatical illusion only when S1 is an embedded clause. An interesting question raised by these findings is whether the same holds for English. Since our account did not appeal to any special properties of German, it predicts that a missing VP1 should also cause a grammatical illusion in an English sentence as in (16), which is identical to the original example of Gibson and Thomas (1999) with the exception that S1 is now an embedded clause.

(16)     I believe that the ancient manuscript that the graduate student who the new card catalog [_VP3_ had confused a great deal] [_VP2_ was studying in the library].

## 6. Experiment 2

In contrast to the SVO languages English and French, all experiments demonstrating a missing-VP effect in German relied on some form of grammaticality judgments, either under time constrained conditions (Bader et al., 2003; Bader, 2015) or without time limitations (Experiment 1). The only study that investigated the missing-VP effect in German using on-line reading measures (selfpaced reading and eye tracking) is Vasishth et al. (2010), and this study failed to find evidence for a grammatical illusion in German whereas it found such evidence for English. Based on the current evidence, it can thus not be excluded that in an SOV language like German a missing-VP effect only occurs when explicit grammaticality judgments are required but not when participants simply process sentences for the purpose of comprehension.

A different possibility is suggested by the results of Bader (2015). These results show that the likelihood of a missing-VP effect in German is modulated by the syntactic configuration in which the center-embedded relative clauses occur. The sentences from Vasishth et al. (2010) contain the relative clauses in the initial position of the main clause whereas the sentences in the current study contain the relative clauses in a sentence-medial position. Using the same speeded grammaticality judgment task as Bader et al. (2003), Bader (2015) found a higher acceptance rate for missing-VP sentences in the latter configuration. The lack of a reading time advantage for missing-VP sentences in the experiments of Vasishth et al. (2010) may thus be due to a weak missing-VP effect in sentences in which the relative clauses belong to a sentence initial NP. If so, we expect that reading time evidence for a missing-VP effect can be found for sentences for which the missing-VP effect is more likely to occur. Experiment 2 tests this prediction by collecting reading times for sentences that, like the sentences investigated in Experiment 1, contain the relative clauses in a sentence medial position.

In the sentences in Experiment 2, S1 is an embedded clause. The sentences are thus structurally similar to the sentences in the “embedded clause” condition of Experiment 1. An example is given in (17). Incomplete sentences were derived by dropping VP2, as indicated by crossing in (17). The subject of S2 is either a singular or a plural NP. The verb of S2, which is only present in complete sentences, is accordingly either a singular or a plural verb. The verb of S1 is always present and always marked for singular in agreement with the subject of S1.

(17)    *Example sentences of Experiment 2*
Ich   glaube
I      think
S1        dass    man     (den    **Direktor**,   /   die   **Direktoren**,)
             that     one       the     principal.sg     the   principals.pl
S2                   (**der**        /   **die**)       den   Schulrat,
                        who.sg       who.pl   the   schools.inspector
S3                           der          das   Projekt  absegnen  soll,
                               who.sg   the    project  approve    should
S2                    alarmiert    (hat,      /   haben,)
                        alarmed      has.sg      have.pl
S1        belogen       **hat**,
             lied.to         has
um  von   dem       eigentlichen   Problem   abzulenken
for   from  the        actual           problem   distract
“I think that the principal who alarmed the schools inspector who was supposed to approve the project was lied to in order to distract him from the actual problem.”

If German was immune to the missing-VP effect, as claimed by Vasishth et al. (2010), reading times should be longer in incomplete sentences compared to complete sentences. The increase should start after the final verb since only then does it become evident that no further verbs are coming and thus a verb is missing. If, on the other hand, the missing-VP effect is present in German too, longer reading times are predicted for the final verb in complete sentences. This prediction is made both by the Pruning Hypothesis and by the Discrimination Hypothesis. Hence, the purpose of Experiment 2 is not to decide between the two hypotheses. Instead, the aim is more modest. The main objective of Experiment 2 is to test whether the missing-VP effect in German can be observed in online measures like reading times at all.

In addition, Experiment 2 tests whether the effect of number reported by Bader et al. (2003) also occurs in on-line reading times. In complete sentences with a plural S2 subject and therefore a plural verb V2, the attempt to integrate V2 into S1 results in a fleeting agreement violation which should increase reading times. Moreover, the integration of the actual verb of S1 then becomes difficult because the verb slot of S1 is already filled by the preceding verb. In incomplete sentences with a plural S2 subject, integration of the final verb, which is always singular, into S2 results in an agreement violation.

### 6.1. Method

#### 6.1.1. Participants

Twenty-four students at the University of Konstanz participated in Experiment 2. They were paid for participation or received course credit.

#### 6.1.2. Materials

We constructed 20 sentences each in four versions. An example is given in (17). All sentences started with a short main clause followed by a complement clause introduced by the complementizer *dass* (“that”). This complement clause (S1) contained an indefinite pronoun as subject and a definite NP as the object followed by a relative clause (S2) modifying the object. The relative clause contained another relative clause (S3), again modifying the object. Due to the clause-final position of verbs in embedded clauses in German, the verbs for S3, S2, and S1 occur in a row after the object of S3. As before, the final position of each clause was filled by a verb cluster consisting of a lexical verb and an auxiliary. The final verb was followed by an adjunct clause in order to minimize wrap-up effects and to provide space for potential spillover effects. Two factors were fully crossed resulting in four conditions. The factor Structure varied whether the sentences were complete or incomplete; in incomplete sentences the intermediate verb (VP2) was omitted. The factor S2-Subject varied the number specification of the head noun of the higher relative clause and thereby of the subject of this relative clause. If present, VP2 matched the S2 subject in number.

For each sentence, we designed a question that probed understanding of the sentence. The example in (18) gives the probe question for (17).

(18)    Hat der Direktor falsche Informationen erhalten?
has the principal wrong information received
“Did the principal receive wrong information?”

As in the example, all probe questions asked for an event involving the subject of S2 which is at the same time the object of S1. Low attachment of VP1 and subsequent interpretation of V1 as the verb of S2 would result in a wrong answer to the probe question. Half of the questions required a positive answer, the other half required a negative answer.

The experimental stimuli were distributed over four lists using a Latin square design. Each participant saw only one list. The order of items in a list was pseudo-randomized for each participant individually. In addition to the experimental stimuli, an experimental session included 94 filler sentences. Most of them served as experimental stimuli in unrelated experiments. Filler sentences were always grammatical and covered a variety of syntactic constructions. The order of filler sentences and experimental items was arranged in such a way that no two experimental items followed each other.

#### 6.1.3. Procedure

Experiment 2 used a word-by-word non-cumulative self-paced reading procedure. Participants read sentences on a computer screen using a moving window display in which all non-space characters of the sentence were initially replaced by underlines (Just et al., 1982). Participants pressed a key on the keyboard to see each new word of the sentence. On each key press, a new word was uncovered and the previous word was again replaced by underlines. The time between successive key presses was recorded automatically. Once the last word of the sentence had been reached, pressing the key again cleared the screen and revealed the word “Frage” (“question”). The next key press produced the question which had to be answered by pushing the “j”-key for “Ja” (“yes”) or the “n”-key for “Nein” (“no”). Participants received no feedback for their answers. To become acquainted with the procedure, participants read four training sentences before the experiment started.

### 6.2. Results

Despite the complexity of the sentences, participants answered probe questions with an overall accuracy of 87%. There were only minimal differences between conditions (range 84–89%). A statistical analysis using a mixed effects model did not find significant effects.

Reading times >2000 ms were removed from the analysis. This affected <1% of the data. The remaining mean reading times are summarized in Table 3. In accordance with Vasishth et al. (2010), we log-transformed raw reading times before fitting linear mixed effects models to the data. Contrasts were coded as follows. The contrast for the factor S2-Subject compares sentences with a singular S2 subject to sentences with a plural S2 subject. The contrast for the factor Structure compares complete sentences to sentences missing the second verb cluster. Fixed effects results for the models are given in Table 4. All models reported in the table contain the full factorial design in the crossed random slopes for participants and items. Since degrees of freedom can only be estimated in linear mixed effects models (Baayen, 2008), we report estimates, standard errors and *t*-values but no *p*-values. An absolute *t*-value of 2 or greater indicates significance at the α-level 0.05. We also computed residual reading times (Ferreira and Clifton, 1986) and repeated all analyses; the results were similar as for the log-transformed raw reading times.

**Table 3:** **Mean reading times in experiment 2**.

**Subject S2**	**Structure**	**VP3**	**VP2**	**VP1**			**Post-VP1**
				**V1**	**Aux1**	**V1 + Aux1**	
Singular subject in S2	Complete	940	1029	544	510	1058	852
Singular subject in S2	Missing	952		481	482	958	829
Plural subject in S2	Complete	928	1038	556	516	1074	844
Plural subject in S2	Missing	934		484	476	951	872

**Table 4:** **Fixed effects of mixed-effect models for reading times in experiment 2**.

	**Structure**			**Subject S2**			**Structure × Subject S2**		
	**Est**.	**SE**	***t***	**Est**.	**SE**	***t***	**Est**.	**SE**	***t***
VP3	−0.008	0.02	−0.34	0.001	0.03	0.03	−0.010	0.05	−0.20
VP2				0.005	0.03	0.13			
VP1	0.090	0.03	3.12	−0.016	0.03	−0.55	−0.003	0.05	−0.06
V1	0.097	0.04	2.76	−0.030	0.04	−0.83	−0.005	0.06	−0.08
Aux1	0.047	0.03	1.45	−0.005	0.03	−0.14	−0.004	0.06	−0.06
Post-VP1	0.005	0.02	0.20	−0.019	0.02	−0.82	0.050	0.04	1.21

For VP3 and VP2, joint reading times for the lexical verb and the auxiliary are virtually identical across conditions (VP3 in sentences with a singular S2 subject: 946 ms, with plural S2 subject: 931 ms; VP2 in sentences with a singular S2 subject: 1029 ms, with plural S2 subject: 1038 ms). The statistical models indicate no significant effect. For VP1, however, reading times are longer in complete sentences (1066 ms in complete sentences, 953 in incomplete sentences). Reading times for individual words reveal that the effect occurs at the lexical verb (550 vs. 483 ms). Numerically, the effect is still visible at the auxiliary but no longer significant (513 vs. 477 ms). At the next word, the effect is gone. The factor S2-subject had no effect at all.

### 6.3. Discussion

The major finding of Experiment 2 is that reading times for the final verb were shorter in incomplete sentences compared to complete sentences. Thus, the missing-VP effect observed in prior judgment experiments occurs as well when participants only have to read for meaning. The difference between the current results and the results of Vasishth et al. (2010) can be attributed to structural differences between the respective sentence materials. As discussed above, the missing-VP effect is weaker when the relative clauses modify an NP in sentence initial position, as in the study of Vasishth et al. (2010). If readers experience a grammatical illusion in only a subset of trials, it may well be that any reading time advantage resulting from trials eliciting a grammatical illusion is offset by a reading time penalty for trials in which readers detect the ungrammaticality.

In contrast to the finding in Bader et al. (2003), the number manipulation had no effect in Experiment 2. We surmise that this difference reflects the fact that Experiment 3 of Bader et al. (2003), but not Experiment 2 of the current study, involved an explicit grammaticality judgment. Since no judgment was required in Experiment 2, the temporary ungrammaticality that might have arisen in conditions with a plural S2 subject could be internally repaired by the parser without any overtly observable effect.

## 7. General discussion

This paper has presented an interference account of the missing-VP effect, that is, the observation that sentences in which a VP is missing can give rise to an illusion of grammaticality. This account is based on experimental investigations of the missing-VP effect in German. While prior reports of the missing-VP effect in German relied on speeded grammaticality judgments, the experiments reported in this paper show that the missing-VP effect is rather robust with regard to the experimental procedure. In particular, the missing-VP effect is so strong that it also occurs when participants have to judge sentences without time pressure, and it occurs as well when participants simply have to read sentences for meaning.

The finding of missing-VP effects in German points to the cross-linguistic generality of this kind of grammatical illusion. It is not confined to languages with SVO order but is found in languages with SOV order too. This suggests that the source of the effect is not language-specific but results from more general mechanisms that apply across languages. Interference during cue-based retrieval is a promising candidate for such a general mechanism. It provides a unified account of how sentences with double center-embedding—whether complete or incomplete—are processed. In sentences with double center-embedding, the parser faces two competing attachment sites for the second verb, as illustrated in (19).

(19)    [_*S1*_ NP1 … [_*S2*_ NP2 … [_*S3*_ NP3 … VP3] … **VP2** … (VP1)

Processing of NP1 causes the creation of a sentence node and thereby leads to the expectation of a verb. Similar expectations result from the processing of NP2 and NP3. Integration of VP3 fills the open verb slot of S3. After processing of S3, the next verb generates retrieval cues that call for a sentence with an open verb slot. Since both S1 and S2 fit this cue, interference arises and hampers the correct integration of the second verb into S2. As a result, the second verb is occasionally integrated into the wrong clause, namely S1, and thereby analyzed as VP1. Erroneous integration of VP2 into S1 entails difficulties for the subsequent integration of VP1 in complete sentences and it contributes to the illusion of grammaticality in missing-VP sentences. To make the illusion perfect, the lack of lexical material in the VP2 slot must go unnoticed. A failure to detect the missing VP is especially likely because the incomplete clause (S2) is no longer the current clause as soon as the parser returns to the higher clause, what it does when attaching the final verb to S1. This reasoning also explains why the status of S1 (main clause vs. embedded clause) had no effect for the likelihood of a missing-VP2 effect. Since the clause lacking VP2 is always an embedded clause, its processing must be completed when the last verb is integrated into the higher clause.

Since nothing is ever deleted according to our account, S1 and S2 are always available as attachment sites and therefore as targets for retrieval. The additional finding of a grammatical illusion when VP1 is missing indicates that the VP slot of S2 is retrieved for integration in some of the cases. In contrast to cases of a missing VP2, a grammatical illusion for a missing VP1 was observed only when S1 was an embedded clause but not when S1 was a main clause. We have argued that this finding is a primacy effect. When S1 is a main clause and thereby occurs in sentence initial position, the probability of erroneously attaching VP2 to it increases as does the probability of detecting that VP1 is missing in case VP2 has correctly been attached to S2. Taken together, this prevents the occurrence of a missing-VP effect for VP1 in main clauses.

Two alternatives to an interference-based account of the missing-VP effect are the resource-based account of Gibson and Thomas (1999) and the experience-based account of Christiansen and MacDonald (2009). The resource-based account of Gibson and Thomas (1999), which was already discussed above, is based on the idea that the parser has only a limited amount of resources available for storage and integration. Their Pruning Hypothesis proposes pruning as a last resort mechanism to free resources and thereby to avoid an overload of the parser. After deletion of VP2, the second verb can only be integrated into S1, creating the illusion of completeness in missing-VP sentences. The assumption of VP2-pruning is disconfirmed by the finding that omitting VP1 can lead to a missing-VP effect as well under certain circumstances. In addition, the Pruning Hypothesis is not attractive from a theoretical point of view. Pruning is a mechanism specific for situations with high memory load and has to be stipulated. Interference, on the other hand, is a general phenomenon that follows from cue-based retrieval. Similarity-based interference arises whenever two or more items in a memory representation are similar to each other. Interference can emanate from an item preceding the target item (proactive interference) or from an item following the target item (retroactive interference). Under the Discrimination Hypothesis, the missing-VP effect is an instance of proactive interference. Interference has been shown to be effective in explaining various phenomena in language comprehension (cf. van Dyke and Johns, 2012; Gordon and Lowder, 2012). We conclude that an interference-based explanation of the missing-VP effect is both empirically and conceptually more adequate than a resource-based explanation.

An experience-based account of the missing-VP effect was proposed by Christiansen and MacDonald (2009). This account draws on earlier work by Christiansen and Chater (1999) who proposed a connectionist model of recursion in natural language. This model is cast as a simple recurrent network (Elman, 1990) that learns from experience to predict the next word of a sentence from the words processed so far. Simulations by Christiansen and MacDonald (2009) show that when processing an English sentence with double center-embedding, the model expects only a single verb after it has encountered V3, as in a missing VP sentence, and not two verbs, as in a corresponding complete sentence. This approach was extended to German by Engelmann and Vasishth (2009). The model that they trained for German predicts that missing-VP sentences do not give rise to a grammatical illusion in German. Based on the experimental evidence from Vasishth et al. (2010), Engelmann and Vasishth (2009) conclude that an experience-based account of the missing-VP effect is superior to a memory-based account (e.g., the Pruning Hypothesis of Gibson and Thomas, 1999) because only the former account predicts that the missing-VP effect is present in English but absent in German.

With regard to the difference between SVO- and SOV-languages, the main thrust of the experience-based account has been succinctly summarized by Vasishth et al. (2010, p. 558): “One consequence of German head-finality is that—due to the relatively frequent occurrence of head-final structures—predictions of upcoming verbs may have more robust memory representations in German than in English. This could result in reduced susceptibility to forgetting the upcoming verb's prediction, even in the face of increased memory load.” As the results of the present study show, this conclusion is premature. When presented with missing-VP sentences, native speakers of German experience a grammatical illusion as well. Furthermore, native speakers also produce such sentences from time to time. In an ongoing analysis of the deWaC corpus^5^, we found a number of authentic missing-VP sentences. A small selection of such examples is provided in Table 5.

**Table 5:** **Authentic examples of the missing-VP effect from the deWac corpus**.

Ebenso	ist	der	Herr	Jesus	Christus,	der	hier	mit	vollem	Titel,	der	Seine	ganze	Größe	und	Herrlichkeit	andeutetet [sic],	Δ,	die	Quelle	von	Gnade	und	Friede.
likewise	is	the	lord	Jesus	Christ	who	here	with	full	title	which	His	whole	grandness	and	glory	indicates,		the	source	of	mercy	and	peace
“Likewise, the lord Jesus Christ who Δ here with full title which indicates His whole grandness and glory is the source of mercy and peace.”																										
Dieser	Typ	entsteht,	wenn	lin-3	oder	ein	Gen,	das	für	die	Induktion,	die	von	der	Ankerzelle	ausgeht,	Δ,	mutiert	ist.
this	type	emerges	when	lin-3	or	a	gene	that	for	the	induction	that	from	the	anchor-cell	originates		mutated	is
“This type emerges when lin-3 or a gene that is Δ for the induction that originates from the anchor cell has mutated.”																										
Dass	wir	hinterfragen,	liegt	schlicht	und	ergreifend	daran,	dass	bis	heute	keine	der	Prognosen,	die	Sie	in	den	Monaten,	die	Sie	im	Amt	sind,	Δ,	eingetroffen	ist.
that	we	question	lies	simply	and	plainly	at-there	that	until	today	none	of-the	predictions	that	you	in	the	months	that	you	in	office	are		happened	is
“That we scrutinize is a simple consequence of the fact that none of the predictions that you Δ during the months that you have been in office has turned out to be true.”																										

Such examples make two points. First, the missing-VP effect is not restricted to language comprehension but occurs in language production as well. Second, the missing-VP effect is not merely a laboratory phenomenon. Since this is evidence from German, we can conclude that the verb-final nature of German does not lead to memory structures that prevent the missing-VP effect from occurring. At face value, this contradicts experience-based accounts which have derived the absence of a missing-VP effect in German from corpus-based simulations. However, drawing strong conclusions at this point would be premature. For example, the training corpus used by Engelmann and Vasishth (2009) for their simulation is not described in detail, which leaves the possibility that their training input did not include all relevant syntactic configurations. Additional simulations are necessary in order to address the issues raised above, but this is beyond the scope of the present paper.

The seeming contradiction between the evidence presented by Vasishth et al. (2010) on the one hand and the evidence provided by Bader et al. (2003) and the analysis of the deWac corpus on the other hand was addressed by Bader (2015). Based on corpus evidence and on evidence from yet another experiment using the method of speeded grammaticality judgments, Bader (2015) showed that the strength of the missing-VP effect varies with the syntactic position occupied by the doubly center-embedded relative clause. The probability that a missing-VP effect occurs is smaller when the relative clauses occupy the initial position of a main clause, as in the sentences investigated by Vasishth et al. (2010) [see (13)], than when they are contained within the lower part of the clause, whether this is an embedded clause as in (14) or a main clause as in (15).

In sum, the results of the present study confirm the existence of the missing-VP effect in German and thereby show that the occurrence of this grammatical illusion does not depend on whether a language is SVO or SOV. The results challenge resource-based and experience-based accounts of the effect, but they lend further evidence to interference-based accounts of human parsing. In particular, the missing-VP effect adds to the existing evidence for proactive interference during language comprehension and supports cue-based parsing architectures.

### Conflict of interest statement

The authors declare that the research was conducted in the absence of any commercial or financial relationships that could be construed as a potential conflict of interest.

## Footnotes

^1^In Gibson and Thomas (1999), VPs are numbered according to their linear position in the sentence string. In later publications (Christiansen and MacDonald, 2009; Gimenes et al., 2009; Vasishth et al., 2010), VPs are numbered according to their hierarchical position in the phrase-structure tree. In this paper, we adopt the latter numbering. VP1 is the VP of the matrix clause (S1), VP2 is the VP of the upper relative clause (S2), and VP3 is the VP of the lower relative clause (S3). The VPs therefore appear in the linear order VP3 VP2 VP1.

^2^Resource-based and interference-based accounts are not necessarily incompatible with each other. For example, cognitive architectures such as ACT-R and its relatives SOAR and 3CAPS usually include both assumptions—a limited amount of resources as well as interference from similar items—but differ with regard to the component they emphasize.

^3^Note that similar assumptions would also be necessary for accounts assuming the deletion of a VP from the phrase structure representation. Pruning of a predicted VP leaves a subject NP without corresponding predicate within the ongoing syntactic structure. Thus, for a missing-VP sentence to be judged as grammatical, a pruning account has to claim that the parser overlooks the dangling subject NP.

^4^For English, Gibson and Thomas (1999) cite an unpublished acceptability experiment by Gibson and Kaan as providing evidence for this claim. In an unpublished experiment using the procedure of speeded grammaticality judgments, we found that German sentences corresponding to (10) were rejected as ungrammatical in almost 90% of the trials.

^5^DeWac is the German part of Wacky, a family of large corpora built by web crawling (Baroni et al., 2009). DeWac contains 1.7 billion tokens of text which is POS tagged and lemmatized (using TreeTagger). Partial results from an ongoing corpus study of complete and incomplete doubly embedded relative clauses in the DeWac corpus can be found in Bader (2015).
